# Recent Advances in Petroleum Microbiology

**DOI:** 10.3390/microorganisms10091706

**Published:** 2022-08-24

**Authors:** Bo-Zhong Mu, Tamara N. Nazina

**Affiliations:** 1 State Key Laboratory of Bioreactor Engineering, School of Chemistry and Molecular Engineering, East China University of Science and Technology, Shanghai 200237, China; 2 Engineering Research Center of MEOR, East China University of Science and Technology, Shanghai 200237, China; 3 Winogradsky Institute of Microbiology, Research Center of Biotechnology of the Russian Academy of Sciences, 119071 Moscow, Russia

Petroleum reservoirs are unique deep-subsurface ecosystems that are generally characterized by such extreme conditions as high temperature, high pressure, high salinity, and anoxia. Microbial communities and their functions in petroleum reservoirs are crucial for a better understanding of the biogeochemical processes and life forms in such an extreme environment. They are also vital for developing new strategies and technologies for microbial enhanced energy recovery (MEER), the control of reservoir souring and corrosion in oil production systems, and the bioremediation of petroleum-contaminated sites. Petroleum reservoirs can be regarded as natural bioreactors and are ideally suited to the study of microbial metabolisms in the deep subsurface. Over the past decades, a combination of culture-dependent and culture-independent studies has highlighted the great diversity of physiologically distinct microorganisms in production waters from oil reservoirs and it certainly advanced our knowledge of subsurface microbial communities and their function. Naturally, the activities of these microorganisms have a significant influence on the geochemistry of oil reservoir environments. Of particular interest is the relationship between microbial metabolic activities and carbon turnover, element cycling, and, probably especially relevant in light of microbial enhanced oil recovery (MEOR), carbon capture, utilization, and sequestration (CCUS) in petroleum reservoirs [1], and the inhibition of microbiologically influenced corrosion (MIC) in oilfields as well.

Microbial enhanced oil recovery technology is of great importance for extracting residual oil from oil reservoirs approaching their economical limit, including oil reservoirs with high water cut, low permeability, and bearing heavy oil that cannot be economically recovered by traditional methods. Extensive studies have been carried out on MEOR regarding the dominant microbial communities in petroleum reservoirs, metabolic products beneficial for enhanced oil recovery, and strategies for oil reservoirs with different conditions. As one of the most cost-effective and environmentally friendly approaches, this technology is promising in terms of involving microbial water flooding, microbial permeability profile modification, and microbial huff and puff. The biosurfactant-induced reduction in oil–water interfacial tensions and the alteration of the wettability of the oil-bearing rocks are the primary mechanisms involved in the microbial water flooding process. The expansion of swept volume caused by biomass accumulation in the high permeability zone of heterogeneous reservoirs plays a key role in microbial permeability profile modification. In past decades, MEOR field trials have been conducted in many oilfields. Most of these field pilot studies have shown positive results in terms of increased oil production and decreased water cut, with an incremental oil recovery rate of approximately 5% of original oil in place (OOIP) and high economic benefits with an input-output ratio of 1:6. Field trials in oil reservoirs with temperatures as high as 91 °C, salinity as high as 46,000 mg/L, and permeability as low as 25 mD have been conducted successfully, indicating the great potential of MEOR technology in applications to different oil reservoirs with harsh physicochemical conditions. Moreover, the microbial in situ degradation of hydrocarbons with methane production may further recover the oil remaining in reservoirs in the form of methane gas, which is a new option to ideally allow all residual oil to be extracted in the form of methane via microbial enhanced energy recovery (MEER).

A variety of microorganisms involved in the bioconversion of CO_2_ to CH_4_ has been revealed in both high- and low-temperature reservoirs by culture-dependent and culture-independent approaches. The functional gene-based analysis of microbial communities in oil reservoirs further confirmed the possibility of CO_2_ conversion pathways, indicating the great potential of the biotransformation of CO_2_ into methane energy in oil reservoirs. Extensive studies on the microbial community and methanogenic biochemical pathways in oil reservoirs have been made in both the laboratory and oilfields. For the bioconversion of CO_2_ to methane in oil reservoirs, certain factors, including microorganisms, electron donors, and their thermodynamic and kinetic behavior, are critical for the methanogenesis process, and among these, the available electron donors appear to be the key factors and limiting steps in the process [2]. Many oil reservoirs have undergone CO_2_ injection for enhanced oil recovery (CO_2_-EOR). A recent study showed that microbial methanogenesis converted as much as 13–19% of the injected CO_2_ to methane in a CO_2_-EOR project in an oilfield based on noble gas, stable isotope, clumped isotope, and gene-sequencing analyses. It also showed that in the course of CO_2_-EOR, additional CO_2_ (up to 74%) was dissolved in the groundwater of the reservoir, indicating that the storage and bioconversion of CO_2_ to methane energy in oil reservoirs are promising approaches to carbon capture, utilization, and sequestration (CCUS).

Members affiliated with sulfate-reducing microorganisms (SRM) and their activities were detected in subsurface petroleum reservoirs and associated oil-well pipelines, where sulfate is present [3]. They are heterotrophic, or autotrophic, anaerobic, or partially O_2_-tolerant microorganisms, which enables them to adapt to a wide range of environmental conditions including temperature, salinity, pressure, etc. Since the first report on petroleum microorganisms showed the presence of sulfate-reducing bacteria in oilfield waters, extensive studies focused on the diversity and composition of sulfate-reducing communities characterized by culture-dependent and/or culture-independent approaches revealed over 220 species of 60 genera of SRM. Among these, some members affiliated with *Proteobacteria, Nitrospira*, *Firmicutes*, *Thermodesulfobacteria*, *Euryarchaeota*, and *Crenarchaeota* were mainly detected in petroleum reservoirs. Sulfate-reducing microorganisms play an important role in the biogeochemical cycles of C, N, and S and choosing a strategy for the exploitation of petroleum reservoirs. Meanwhile, SRM can oxidize H_2_ or organic matter (crude oil hydrocarbons, volatile fatty acids, alcohols, aromatic compounds, etc.) accompanied by a series of sulfate reduction processes to H_2_S, and their activities imply their potential contributions to MIC, reservoir souring, and crude oil biodegradation in petroleum reservoir systems. Various inhibition strategies involving the injection of biocides or other inhibitors (such as nitrate, perchlorate, etc.) have been applied to mitigate souring and biocorrosion in oilfields. Depending on the structure of the SRM community and the functional mechanisms operating in different oil reservoirs, it is of great value to select specific inhibition strategies to deal with reservoir souring and MIC.

The above-mentioned problems of petroleum microbiology are considered in a Special Issue “Petroleum Microbiology”.

Three articles of the Special Issue are devoted to the study of sulfidogenic prokaryotes from oil reservoirs, the suppression of sulfide formation, and the corrosion of steel equipment [4,5,6]. Davidova and co-workers were among the few researchers who identified sulfate-reducing bacteria capable of utilizing *n*-alkanes. These bacteria are capable of the single-stage degradation of oil by reducing sulfate to sulfide and do not depend on microorganisms of the previous trophic level. At present, there are several dozens of strains that can use alkanes under anoxic conditions. The sulfate-reducing strain *Desulfoglaeba alkanexedens* ALDC is one of these unique strains. All the works related to the study of the role of this bacterium in the degradation of oil and the corrosion of steel equipment are of great value. The study by Davidova et al. [4] demonstrated the ability of the *Desulfoglaeba alkanexedens* strain ALDC to produce extracellular polymeric substances (EPS) when growing on a decane-amended medium and preliminarily characterized the EPS composition. It was shown that the EPS of this organism was not particularly corrosive and could protect the metal from oxidation. The metabolic end products of the hydrocarbon metabolism of *D. alkanexedens* are more important factors involved in steel corrosion than the biofilm matrix, which may facilitate microbial access to hydrocarbon substrates in an oil–water interface.

The study by Sokolova et al. [5] demonstrated the influence of seawater injection on the microbial community of the Uzen high-temperature petroleum reservoir located in Kazakhstan. The penetration of seawater resulted in the temperature decrease in the near-bottom zone of injection wells and the increase in sulfate in the formation water, changing the microbial composition in favor of sulfidogenic prokaryotes and sulfide production. In laboratory experiments, it was shown that the use of nitrate to suppress the growth of sulfidogens in a mesophilic enrichment was ineffective since mesophilic denitrifying bacteria reduced nitrate to dinitrogen, which confirmed the observations made at other oilfields. However, thermophilic denitrifying enrichments from the Uzen oilfield produced up to 2.2 mM of nitrite, an inhibitor of sulfate-reducing bacteria (SRB). Sulfate- and thiosulfate-reducing enrichments and a pure culture of SRB *Desulfovibrio alaskensis* Kaz19, isolated from the oilfield, formed biofilms highly resistant to biocides. These results show that seawater injection and the temperature of the environment determined the prokaryotic composition and functional activity in the Uzen oilfield. The authors stressed that the ability of *D. alaskensis* to form biofilms and extracellular microbial nanowires and to produce molecular hydrogen in the absence of sulfate makes it a possible participant of transmembrane electron transfer and an ecologically valuable component of subterranean sulfidogenic communities.

Nicoletti et al. [6] demonstrated the real effect of nitrate injection on the composition of the microbial community of formation water from the high-temperature oilfield located in the Joan d’Arc basin approximately 300 km off the coast of Eastern Canada. The water samples were collected from the topside infrastructure vessels at two platforms (A and B) that process the fluids during offshore oil production operations. Seawater injection was used for secondary oil recovery. Platform A represented an oil recovery operation not receiving nitrate treatment for souring, while the reservoir fluids processed by Platform B had received nitrate treatment. Water samples from various topside vessels were amended with nitrate, nitrite, or/and sulfide, incubated at 54 °C, and the microbial community composition and sulfate reduction rates were compared with untreated water samples. These microcosm tests revealed that the platform B microbial communities responded to nitrate amendment by an increase in nitrate-reducing activities, the production of up to 2.45 mM nitrite, and the inhibition of sulfate reduction. In contrast, nitrate treatments were not effective in preventing sulfide production by microcosms from Platform A. Nitrite amendment had the strongest sulfate reduction inhibition in the samples from both platforms but exhibited the highest pitting density. These results provide evidence of high risk for the microbial corrosion of topside vessels during offshore oil production operations from this high-temperature oilfield.

The study by Scheffer et al. [7] describes the results of the characterization of the microbial diversity of a moderately high-temperature petroleum reservoir (52 °C) in the Gulf of Mexico which contained highly saline formation water. This oilfield is exploited without water-flooding. The microbial diversity, assessed by 16S rRNA gene-based sequencing, revealed only 10 OTUs from seven genera in the formation water and enrichments. Shotgun metagenomic sequencing was performed to better understand the physiology and genomic potential of organisms living in the reservoir. Nine metagenome-assembled genomes (MAGs) were affiliated with the genomes of members of the genera *Arhodomonas*, *Flexistipes*, *Geotoga*, and *Marinobacter*, and of the novel lineages QPJE01 (genus level) within the *Halanaerobiaceae* and BM520 (family level) within the *Bacteroidales*. Genomic analysis provided evidence for the adaptation of these bacteria to high salinity and radioactivity, resistance to metals, and the potential for quorum sensing and biofilm formation. The potential for dissimilatory nitrate reduction and denitrification and the ability to reduce nitrate to nitrite were revealed in the MAGs of *Arhodomonas*, *Flexistipes*, *Geotoga*, and *Marinobacter*, which may be favorable for the suppression of sulfidogenesis.

The article by Hidalgo et al. [8] presents the results of the analysis of a huge array of data of the 301.2 Gb of metagenomics information derived from water-flooded and non-flooded petroleum reservoirs with various temperatures located in China, Alaska, and Brazil. These authors reconstructed a total of 148 metagenome-assembled genomes (MAGs) and analyzed the potential functional core in the reservoirs using the Kyoto Encyclopedia of Genes and Genomes (KEGG) database. It was shown that 1690 potential functional core genes, representing 31.7% of the total functional annotated genes, were common to all oilfields, while 1007 genes (18.8% of the total annotated KEGG orthologies) were site-specific genes occurring only in one of the oilfields. The oil reservoirs with a lower level of intervention were the most similar to the potential functional core, while the water-flooded oilfields had greater variation in the functional profiles.

In the research of Gilbert et al. [9], hydrocarbon biodegradation in the Tokamachi mud volcano (Japan) was detected. Using a combination of data on the position-specific ^13^C isotope composition of propane, chemical, and 16S rRNA gene sequence analyses, it was shown that in the studied samples, propane and other C_2+_ hydrocarbons were oxidized by anaerobic organisms with indirect methane production by hydrogenotrophic and acetoclastic methanogens. Anaerobic propane-oxidizing prokaryotes need further research.

The diversity of prokaryotes involved in anaerobic oil degradation in oilfields deserves much attention. The paper by Semenova et al. [10] describes the composition of an anaerobic oil-degrading methanogenic enrichment obtained from a heavy oil reservoir (Russia). Using a 16S rRNA-based approach, it was shown that the prokaryotic diversity of the enrichment was low and consisted of hydrogenotrophic methanogenic archaea of the genus *Methanobacterium*, anaerobic bacteria of the genera *Sedimentibacter* and *Paeniclostridium*, and facultatively anaerobic organotrophic bacteria of the genera *Actinotalea*, *Pseudomonas*, *Tepidimonas*, and *Rhodoferax*. Anaerobic growth of enrichment on crude oil resulted in the accumulation of H_2_ and CH_4_ in the gas phase and of acetate in the medium. The facultatively anaerobic *Actinotalea* strains HO-Ch2^T^ and HO-62b1, isolated from the enrichment, were described as a new species, *Actinotalea subterranea* sp. nov. The suggested ecophysiological function of these bacteria is the fermentation of carbohydrate- and protein-containing substrates, including dead biomass, with the generation of carbon sources (e.g., acetic, propionic, iso-butyric, iso-valeric acids, and CO_2_), and the recycling of other nutrients (e.g., N and P), which at the terminal stage of oil biodegradation are used by methanogenic members of the community.

Thermal methods are most often used for the development of heavy oil deposits. After the end of their application, the temperature of an oil reservoir decreases back to its original values. The possibility of the application of microbial enhanced oil recovery methods in such areas of oil reservoirs was considered in the article by Hu et al. [11]. In laboratory conditions, the authors simulated the thermal treatment of an oilfield by means of single- or double-autoclaving of oil from the Daqing field. Using DNA- and RNA-based approaches, the composition of the total and metabolically active microbial communities originally present in oil and surviving after the thermal oil treatment and the biodegradation of the oil was determined. The effect of the introduced strains of the hydrocarbon-oxidizing bacteria *Amycolicicoccus subflavus* DQS3-9A1^T^ (reclassified as *Hoyosella subflava*) and *Dietzia* sp. DQ12-45-1b on the composition of the oil community and the oxidation of oil was also evaluated. The *Dietzia* sp. strain more effectively induced the proliferation of varied species in one-time heated crude oil than *A. subflavus* DQS3-9A1^T^. The results obtained indicate that some reservoir microorganisms can remain viable after thermal treatment, and the introduction of hydrocarbon-oxidizing bacteria adapted to the conditions of the oil reservoir, degrading oil with the formation of biosurfactants and acidic products, can be used as a MEOR method in thermal recovery-processed oil reservoirs.

The review by Li et al. [12] summarizes the extensive literature and the authors’ results of the study of biosurfactants formed under anaerobic conditions by the fifty-eight reported bacterial strains, mostly isolated from oil reservoirs. Special attention is paid to the species *Bacillus subtilis*, producing lipopeptide biosurfactants, and the species *Pseudomonas aeruginosa*, producing glycolipid biosurfactants. These strains demonstrated an ability to produce biosurfactants under anaerobic conditions, but the biosurfactant yield is much lower than that under aerobic conditions. Thus, the metabolic pathway of biosurfactant-producing bacteria under anaerobic conditions is still a challenge for developing a strategy to further promote the biosurfactant yield in such conditions. It is also important for the applications of biosurfactant-producing bacteria in bioremediation, MEOR, and oxygen-limiting environments.

The editors hope that original articles and review published in this Special Issue will be useful for researchers interested in petroleum microbiology and biotechnology.

## References

[1] Tyne R.L., Barry P.H., Lawson M., Byrne D.J., Warr O., Xie H., Hillegonds D.J., Formolo M., Summers Z.M., Skinner B. (2021). Rapid microbial methanogenesis during CO_2_ storage in hydrocarbon reservoirs. Nature.

[2] Kazmi M., Irfan M., Zhou L., Yuan S., Fatima H., Tian L.-Y., Ye Y.-L., Lu Q.-S., Lu X.-Y., Yang S.Z. (2022). Electron donors and mediators in the thermodynamics and kinetics of CO2 bioreduction. Renew. Sustain. Energy Rev..

[3] Marietou A., Gadd G.M., Sariaslani S. (2021). Chapter Two—Sulfate reducing microorganisms in high temperature oil reservoirs. Advances in Applied Microbiology.

[4] Davidova I.A., Lenhart T.R., Nanny M.A., Suflita J.M. (2021). Composition and Corrosivity of Extracellular Polymeric Substances from the Hydrocarbon-Degrading Sulfate-Reducing Bacterium *Desulfoglaeba alkanexedens* ALDC. Microorganisms.

[5] Sokolova D.S., Semenova E.M., Grouzdev D.S., Bidzhieva S.K., Babich T.L., Loiko N.G., Ershov A.P., Kadnikov V.V., Beletsky A.V., Mardanov A.V. (2021). Sulfidogenic Microbial Communities of the Uzen High-Temperature Oil Field in Kazakhstan. Microorganisms.

[6] Nicoletti D., Sharma M., Gieg L.M. (2022). Assessing Microbial Corrosion Risk on Offshore Crude Oil Production Topsides under Conditions of Nitrate and Nitrite Treatment for Souring. Microorganisms.

[7] Scheffer G., Hubert C.R.J., Enning D.R., Lahme S., Mand J., de Rezende J.R. (2021). Metagenomic Investigation of a Low Diversity, High Salinity Offshore Oil Reservoir. Microorganisms.

[8] Hidalgo K.J., Sierra-Garcia I.N., Zafra G., de Oliveira V.M. (2021). Genome-Resolved Meta-Analysis of the Microbiome in Oil Reservoirs Worldwide. Microorganisms.

[9] Gilbert A., Nakagawa M., Taguchi K., Zhang N., Nishida A., Yoshida N. (2022). Hydrocarbon Cycling in the Tokamachi Mud Volcano (Japan): Insights from Isotopologue and Metataxonomic Analyses. Microorganisms.

[10] Semenova E.M., Grouzdev D.S., Sokolova D.S., Tourova T.P., Poltaraus A.B., Potekhina N.V., Shishina P.N., Bolshakova M.A., Avtukh A.N., Ianutsevich E.A. (2022). Physiological and Genomic Characterization of *Actinotalea subterranea* sp. nov. from Oil-Degrading Methanogenic Enrichment and Reclassification of the Family *Actinotaleaceae*. Microorganisms.

[11] Hu B., Zhao J.-Y., Nie Y., Qin X.-Y., Zhang K.-D., Xing J.-M., Wu X.-L. (2021). Bioemulsification and Microbial Community Reconstruction in Thermally Processed Crude Oil. Microorganisms.

[12] Li J.-Y., Wang L., Liu Y.-F., Zhou L., Gang H.-Z., Liu J.-F., Yang S.-Z., Mu B.-Z. (2021). Microbial Lipopeptide-Producing Strains and Their Metabolic Roles under Anaerobic Conditions. Microorganisms.

